# CHOT ACADEMIC PARTNER

**DOI:** 10.1093/geroni/igz038.1547

**Published:** 2019-11-08

**Authors:** Robert Weech-Maldonado

**Affiliations:** University of Alabama at Birmingham, Birmingham, Alabama, United States

## Abstract

The second speaker will be Dr. Robert Weech-Maldonado, an academic representative and CHOT Site Director at the University of Alabama at Birmingham. Dr. Weech-Maldonado will share what got UAB involved in CHOT; the process to get involved; what it means to be a CHOT partner, and the pros/cons of this program from the academic side. During this symposium, we will explore why this model needs to be expanded to the field of gerontology and the impact it can have on the aging population. Relating to the academic experience, Dr. Maldonado will speak to how CHOT can result in financial support to the academic institution; provide access to unique data sets / populations; outcomes, such as, publications, and how this program can further academics careers. We will also explore some of the struggles related to working with an industry partner as it relates to deliverables and expectations.

